# Development and Concordance of Binding and Neutralizing Assays to Determine SARS-CoV-2 Antibody Activity in Human Milk

**DOI:** 10.20411/pai.v10i2.799

**Published:** 2025-05-22

**Authors:** Mallory C. Shriver, Patricia L. Milletich, Alberto Moreno, Sasha E. Larsen, Christine M. Posavad, Bryan J. Berube, Bushra Wali, Madison Ellis, Kelly Manning, Kathryn M. Moore, Zhiyi Zhu, Nimrit Grewal, Ines A. Cadena, Cristina V. Cardemil, Flor M. Munoz, Kathleen M. Neuzil, Rhea N. Coler, Mehul S. Suthar, Marcela F. Pasetti, the MOMI-Vax Study Group

**Affiliations:** 1 Center for Vaccine Development and Global Health, University of Maryland School of Medicine, Baltimore, Maryland; 2 Center for Childhood Infections and Vaccines, Children’s Healthcare of Atlanta, Division of Infectious Diseases, Department of Pediatrics, Emory Vaccine Center, Emory University, Atlanta, Georgia; 3 Emory National Primate Research Center, Atlanta, Georgia; 4 Division of Infectious Diseases, Department of Medicine, Emory University School of Medicine, Atlanta, Georgia; 5 Seattle Children’s Research Institute, Center for Global Infectious Disease Research, Seattle, Washington; 6 Vaccine and Infectious Disease Division, Fred Hutchinson Cancer Center; Department of Laboratory Medicine and Pathology, University of Washington, Seattle, Washington; 7 National Institute of Allergy and Infectious Diseases, National Institutes of Health, Rockville, Maryland; 8 Departments of Pediatrics and Molecular Virology & Microbiology, Baylor College of Medicine, and Texas Children’s Hospital, Houston, Texas; 9 University of Washington School of Medicine, Department of Pediatrics, Seattle, Washington; 10 University of Washington Department of Global Health, Seattle, Washington; 11 Division of Infectious Diseases, Department of Pediatrics, Emory University School of Medicine, Atlanta, Georgia

**Keywords:** SARS-CoV-2 Antibodies, Breast Milk, Multiplex Assays, SARS-CoV-2 Neutralization

## Abstract

**Background:**

Maternal immunization provides vaccine-specific immunity to the infant via breast milk. Multiple studies have reported the presence of SARS-CoV-2 antibodies in human breast milk (HBM) from infected and/or vaccinated women. However, there is limited information on the analytical performance, consistency, and quality of the methods used. Standardized and rigorous assays are needed to meet clinical study endpoints and for comparisons across studies.

**Methods:**

We optimized high-throughput multiplex immunoassays for quantification of SARS-CoV-2 immunoglobulin (Ig)G and IgA in HBM and determined antibody levels in HBM samples from 236 SARS-CoV-2 vaccinated (infected and non-infected) and 50 pre-pandemic (unexposed) lactating women. Additionally, SARS-CoV-2 neutralizing activity was examined in a subset of 75 SARS-CoV-2 HBM from vaccinated (infected and non-infected) women using live virus focus reduction neutralization and pseudovirus assays. Concordance between SARS-CoV-2 binding and live virus neutralization outcomes was examined.

**Results:**

The multiplex SARS-CoV-2 assays had adequate analytical sensitivity, repeatability, precision, and assay linearity and were reliable for quantification of IgG and IgA in HBM. Positivity thresholds for Spike- and Nucleocapsid-specific IgG and IgA were established; IgG discriminated positive/negative SARS-CoV-2-immune HBM with high sensitivity and specificity, while IgA reactivity overlapped. A strong correlation was observed between live SARS-CoV-2 and pseudovirus neutralization activity. HBM Spike IgA and neutralization titers were highly correlated.

**Conclusions:**

SARS-CoV-2 binding and neutralizing antibody activity in HBM was determined using standardized and rigorous assays. HBM positivity cutoff values for SARS-CoV-2 vaccination and infection were established. The methods and approach described here could be applied to other pathogens and mucosal secretions.

## INTRODUCTION

Maternal immunization reduces the risk of SARS-CoV-2 infection and hospitalization of both mothers and infants in the first months of life [1–3], and therefore, COVID-19 vaccines are recommended at any time point during pregnancy and postpartum [4].

Maternal and placental transfer of SARS-CoV-2 humoral immunity has been extensively characterized [5]. However, the impact of vaccination and infection during pregnancy and lactation on mucosal immunity available to infants via breastfeeding remains poorly understood.

While placentally acquired antibodies wane, maternal milk remains the sole source of pathogen-specific immunity until infants are eligible for COVID-19 vaccination at 6 months of age [1–3]. Breastfeeding is known to reduce the incidence and severity of respiratory and enteric infections and is recommended to promote child health and survival [6, 7].

Human breast milk (HBM) is rich in immunologically active biomolecules, particularly secretory immunoglobulin (Ig) A (sIgA) [8]. Mucosal IgA has been associated with a lower risk of SARS-CoV-2 variant infection [9, 10] and faster viral clearance and symptom resolution [11]. Host defense mechanisms capable of blocking SARS-CoV-2 in the respiratory mucosa are needed for early control of infection and to slow or block transmission [9, 12, 13]. The licensed mRNA COVID-19 vaccines elicit strong systemic IgG responses, including neutralizing antibodies, but limited mucosal immunity [13, 14].

Multiple studies have documented the presence of SARS-CoV-2 antibodies in HBM in response to vaccination and/or infection [5, 13, 15, 16]. Some have also examined the capacity of HBM to neutralize virus activity [17–19]. Unfortunately, the lack of standardized methods for the quantification of antibodies in HBM hinders the comparison of data across studies [5, 20]. Importantly, there is little to no information on the stage of development and bioanalytical characterization of assays used that would demonstrate their quality and consistency. There is a need for standardized, rigorous, and reliable methods that could be used to support clinical study endpoints and allow for comparisons across studies.

Here, we describe the development and optimization of high-throughput, multiplexed electro-chemiluminescence immunoassays (ECLIAs) using Meso Scale Discovery (MSD) V-PLEX kits. Assay performance was evaluated by assessing sensitivity, precision, dilutional linearity, parallelism, and matrix effects. The ECLIAs were applied to determine IgG and IgA content in a subset of HBM samples collected under the Multisite Observational Maternal and Infant Study for COVID-19 (MOMI-Vax) study, National Institute of Allergy and Infectious Diseases (NIAID) Division of Microbiology and Infectious Diseases (DMID) protocol 21-0004 (ClinicalTrials.gov Identifier: NCT05031468). SARS-CoV-2 Spike and (nucleocapsid) N positivity cutoff values were established by comparing antibody levels in pre-pandemic as well as HBM samples from mothers enrolled in the MOMI-Vax study who had been vaccinated and self-reported COVID-19 infections. SARS-CoV-2 neutralization activity was also evaluated in the selected subset of MOMI-Vax HBM samples using pseudovirus and live-virus neutralization assays, and concordance between binding and functional antibody readouts was examined.

## METHODS

### Human Breast Milk Samples

To develop the multiplex binding antibody (bAb) assays, 50 pre-COVID-19 pandemic HBM samples were obtained from the DMID protocol 09-0007 (ClinicalTrials.gov Identifier: NCT01181323). Briefly, this study began in 2011 and was completed in 2013; it collected HBM from lactating mothers in 6 locations across the United States as part of an influenza vaccine trial [21]. Convenience (anonymized) samples were also obtained from various academic sites for use as controls and in assay development testing.

For binding and neutralization assay concordance analysis, a subset (n=75) of positive HBM samples was selected from lactating women participating in the MOMI-Vax (21-0004) protocol. This study enrolled 240 women who received COVID-19 mRNA vaccines during pregnancy (2 or 3 doses at least 14 days prior to delivery) or postpartum from July 2021 to January 2022 [22]. The subset of specimens selected included HBM with anti-Spike IgG or IgA titers ≥100 arbitrary units (AU)/mL (measured as described below); 20 of these samples were among the 10% with the highest IgG and IgA anti-Spike titers from all study samples, 15 had the highest IgG titers, 15 had the highest IgA titers, and 25 had both IgG and IgA titers between 100 and 1000 AU/mL. All measurements (bAb, pseudovirus neutralization, and live-virus neutralization) were conducted on HBM that had been defatted by centrifugation at 400xg for 20 minutes at 4°C; the top lipid layer was removed, and the aqueous supernatant was collected and stored at -80°C until use. Binding antibody data from 236 HBM samples tested for the MOMI-Vax study, available as part of the per-protocol testing, were leveraged to determine SARS-CoV-2 Spike and N bAb positivity cutoff values. HBM samples were collected between October 2021 and November 2022, spanning the SARS-CoV-2 Delta to Omicron variant transition.

### Ethics

Clinical samples used in the study were obtained from individuals enrolled in approved protocols who provided informed consent. Anonymized samples were provided to the endpoint laboratories (University of Maryland, Emory Vaccine Center, and Seattle Children’s Research Institute) for testing.

### SARS-CoV-2 Multiplexed Binding Antibody Assay

#### Selection of optimal assay conditions

The V-PLEX SARS-CoV-2 Panel 2 IgG and IgA kits (MSD) used for bAb assays consisted of multi-spot 96-well plates coated by the manufacturer with SARS-CoV-2 (Wuhan strain) Spike protein, S1 subunit Receptor Binding Domain (RBD), and N protein. The assay was conducted following the basic protocol recommended by the manufacturer [23], which was modified and optimized for quantification of bAb in HBM. Various combinations of reagents (blocking buffer, diluent solution, and detection antibody) and conditions (number of washes, blocking incubation time, and shaking speed) were tested across several plates. The kit-included blocking buffer and diluent solution were compared against 10% nonfat dry milk (NFDM; Nestlé) in phosphate-buffered saline (PBS; Quality Biological) pH 7.4 containing 0.05% Tween-20 (Sigma-Aldrich) (PBST+NFDM) solution (for both blocking and diluting). For measurement of IgA, the detection antibody included in the kit was compared against the SULFO-TAG Conjugated Anti-Hu/NHP IgA Antibody (MSD). The suitability of protocols was assessed by evaluating the electrochemiluminescent (ECL) signal-to-noise ratio and the difference between SARS-CoV-2 bAb negative (ie, historic, pre-pandemic) and positive (ie, post-vaccination and/or infection) HBM samples. Assay conditions were selected as those that produced the lowest non-specific binding, best discrimination of positive vs negative samples, and parallelism between the reference standard and test samples. Once the optimal conditions were established, experiments were conducted to characterize assay suitability for determining bAb levels in the HBM matrix. The multiplex ECLIAs were optimized following guidelines from NIAID DMID Regulatory Affairs Assay Development/Qualification/Validation Requirements for Nonclinical and Clinical Immunological Assays (2017) and US FDA Bioanalytical Method Validation Guidance for Industry [24].

Assay steps in brief: SARS-CoV-2 Panel 2 plates were blocked with 150 µL/well of PBST+NFDM solution for 30-120 minutes; a minimum of 30-minute blocking time is recommended by manufacturer; 30-120 minutes yielded no differences in ECL signals [23]. All incubations were performed at 22°C in an incubator (20-26°C recommended by the manufacturer), and plates were agitated on an orbital microplate shaker at 700 rotations per minute (rpm). After blocking, plates were washed 3 times with 150 µL/well PBST. Samples and reference standards were diluted in PBST+NFDM. The reference material provided in the kit was 4-fold serially diluted to generate a 7-point standard curve. Diluted HBM samples, positive and negative controls, the reference standard, and a diluent-only blank were added to plates in duplicate (50 µL/well). Plates were incubated for 2 hours and were subsequently washed as described above. Specific IgG was detected with MSD SULFO-TAG Anti-Human IgG diluted (1X) in PBST+NFDM. Likewise, IgA was detected with MSD SULFO-TAG Conjugated Anti-Hu/NHP IgA diluted to 1.0 µg/mL PBST+NFDM. The appropriate detection antibody was added to all wells at 50 µL/well. After a 1-hour incubation and a final wash, MSD GOLD Read Buffer B was added to plates (150 µL/well). Plates were immediately read on the MSD QuickPlex SQ 120MM reader and analyzed with the MSD Methodical Mind software. ECL signals emitted by the detection antibody for each spotted antigen were analyzed using MSD Discovery Workbench Version 4.0 software. The standard curves for each antibody specificity were fitted with a 4-parameter logistic (4PL) regression model with 1/Y^2^ weighting, and bAb concentrations in the diluted samples were interpolated.

#### Definition of standard curve, parallelism, dilutional linearity

Reference Standard 1 (RS1) (MSD), a serum-based reagent with assigned anti-SARS-CoV-2 IgG and IgA concentrations, was used to calculate bAb titers. RS1 was calibrated by the manufacturer against the first WHO International Standard for anti-SARS-CoV-2 Immunoglobulin (NIBSC code: 20/136)[25], thereby allowing reporting of antibody levels in binding antibody units (BAU/mL). RS1 ECL signals were analyzed using 4PL regression analysis as described above. The goodness of fit of the 4PL model was evaluated by coefficient of determination (R^2^). The dose-response curves of the reference material and those of serially diluted samples in binding assays must be parallel to support the assumption that bAb features are sufficiently similar and allow interpolation of antibody concentrations of an experimental sample into the standard curve [26]. Binding patterns of serially diluted RS1 and HBM samples were examined by comparing linear regression curves of interpolated antibody concentration (BAU/mL) vs dilution factor (both axes log-transformed). The slopes of the linear regressions were compared for each assay using Analysis of Covariance, and the *P*-value (2-tailed) was calculated to test the null hypothesis, ie, no difference between RS1 and HBM binding features.

Additionally, antibody concentrations obtained from serially diluted HBM samples were used to assess assay linearity. The linearity of an immunoassay reflects its ability to generate results that are directly proportional to the concentration of the analyte in the diluted sample [27]. To assess assay linearity, the log-transformed bAb concentrations obtained for each dilution tested were plotted vs the log-transformed expected concentration. Slopes (expected to be 0.8-1.2, with a slope of one indicating a perfectly linear relationship between anticipated and obtained antibody concentration), R^2^ (≥ 0.98), and standard deviation (SD) of the residuals (Sy.x) were examined.

#### Matrix effect and limits of quantification

Constituents of the biological matrix may interfere with the detection of the target analyte [28, 29]. These interferences, called matrix effects, result in non-specific binding that can alter antibody measurements [28]. To reduce the matrix effect, a minimum required dilution (MRD), the lowest dilution (most concentrated) at which a sample may be tested with sufficient accuracy, was determined. HBM samples with relatively low bAb reactivity (n=4) were 2-fold serially diluted, neat or starting at 1:2 dilution, and bAb concentrations were interpolated from the standard curve. For each analyte, matrix effects were examined by calculating %Linearity ([concentration × dilution factor] / [previous concentration in dilution series × dilution factor] × 100). The MRD was selected as the lowest dilution factor, at which 3 out of 4 samples per assay demonstrated acceptable dilutional linearity (80% to 120%). The manufacturer established the lower limit of detection (LLOD) of RS1 for each assay. The lower limit of quantification (LLOQ), the minimum bAb concentration that could be reliably quantified in HBM, was determined as the product of the LLOD and the MRD. The detection ranges (linear regions) for each 4PL standard curve were automatically generated by MSD Workbench.

#### Assay controls and precision

HBM controls were identified and included in each assay to ensure consistency. Two positive control samples with mid-to-high IgG and IgA reactivity against SARS-CoV-2 antigens were obtained from lactating women with a history of COVID-19 infection and vaccination. A commercially available single-donor HBM sample (Innovative Research, Inc.) procured prior to the pandemic was used as a low-negative control sample. Each control HBM was tested by 2 independent operators over 2 days, generating 50-60 replicates; mean values were calculated and designated as the bAb concentrations of each control. These tests were also used to evaluate precision, ie, the agreement of results from separate tests. Intra-assay precision concerns the closeness of results within-run or within-day (ie, all factors held constant) [30] and was assessed by calculating the % coefficient of variation (CV) between replicate results from one plate. Inter-assay (or intermediate) precision measures the variability of results between runs or days [30]. The %CV between results from independent operators over 2 days was determined. The acceptable intraand inter-assay precision threshold was ≤20% CV [24].

#### Quality control

A set of system suitability criteria (SSC) was established to monitor and affirm assay performance. The standards, 3 control samples, and blank wells were included in duplicate on each plate. Recovery of each point in the standard and the controls was required to fall between 70% and 130% of the assigned values, as recommended by the manufacturer (except for the IgG low/negative control). The SARS-CoV-2 IgG reactivity of the negative control was below the LLOD; therefore, ECL signals, instead of concentrations, were averaged, and upper ECL limits were set at 3 standard deviations above the mean.

Furthermore, duplicate wells of controls had to demonstrate a %CV ≤25%. Variation in the standards was limited to %CV ≤20% or ≤25% if near/below the LLOD, and the R^2^ was required to be ≥0.98. The maximum allowable ECL signals of blank wells were also defined. If any of these criteria were not met, the data were rejected, and the assay plate was rerun. Lastly, samples were required to be tested in duplicate, to have produced signals between the detection limits and have %CVs ≤25%. If the ECL was outside the detection limits, the sample was repeated at an appropriate dilution, but not below the MRD.

#### SARS-CoV-2 Spike and Nucleocapsid IgG and IgA positivity cutoff

SARS-CoV-2 Spike bAb cutoff levels were established to discriminate between HBM positive and negative samples, whereas SARS-CoV-2 N bAb cutoff levels were established to discriminate between HBM infection-derived positive and negative samples. Spike and N IgG and IgA titers (BAU/mL) in HBM from women never exposed to (pre-pandemic) SARS-CoV-2 or from women vaccinated (with or without infection) were classified using receiver operating characteristic (ROC) and Area Under the ROC Curve (AUC) analysis. To establish N cutoff values, HBM from vaccinated±infected individuals were segregated based on self-reported COVID-19 infection. Data were log-transformed, and the optimal cutoff values were identified as the points at which AUC was largest (closest to 1), indicating the greatest sensitivity and specificity.

### Neutralization Assays

#### Focus Reduction Neutralization Test (FRNT)

VeroE6-TMPRSS2 cells were generated and cultured as previously described [31]. Two viruses were used in the FRNT: 1) SARS-CoV-2 D614G variant (SARS-CoV-2/human/USA/GA-EHC-083E/2020. Accession ID: EPI_ISL_454690) isolated from residual nasopharyngeal swabs from a patient in Atlanta, GA and 2) a recombinant infectious clone derived from the SARS-CoV-2 D614G variant expressing enhanced green fluorescent protein (eGFP). Variants were plaque purified and propagated once in VeroE6-TMPRSS2 cells to generate working stocks. Representative stock aliquots were deep-sequenced and confirmed as previously described [32].

Live FRNT assays applied to HBM following procedures described for serum and plasma [31–33] except that samples were tested starting neat. They were then 3-fold serially diluted, and an equal volume of SARS-CoV-2 D614G or SARS-CoV-2 D614G-eGFP was added (100-200 PFU). Plates were incubated at 37°C for 1 hour, and the wells containing the immune complexes were loaded onto a VeroE6-TMPRSS2 cell monolayer and incubated at 37°C for an additional hour. Following incubation, the cell supernatant was removed and replaced with a 0.85% methylcellulose (R&D Systems, Inc.) overlay. Plates were incubated at 37°C for 16 hours and then removed, washed, and cells were fixed with 2% paraformaldehyde. Cells incubated with SARS-CoV-2 D614G were permeabilized and incubated with Alexa Fluor-647-conjugated SARS-CoV-2 antibody (AF647-CR3022, sourced from Dr. Jens Wrammert; Emory University, Atlanta, GA) for 4 hours at room temperature or 4°C overnight. After incubation, cells were washed, placed in Dulbecco’s PBS, and visualized on an ELISpot reader (CTL Analyzer). Cells incubated with SARS-CoV-2 D614G-eGFP were directly visualized on an ELISpot reader without permeabilization. Antibody neutralization was quantified by counting the number of foci in each well using the Viridot program (https://github.com/leahkatzelnick/Viridot) [34]. The percent of virus neutralization was calculated as = 100-([mean number of foci in HBM/mean number of foci at the highest dilution for the same HBM sample] x100]). Each specimen was tested in duplicate. The FRNT_50_ titers (the reciprocal dilution of HBM that neutralizes 50% of the input virus) were interpolated using a 4PL regression in GraphPad Prism 10.3.1. Samples that did not achieve 50% neutralization were plotted at 2.

#### Pseudovirus neutralization

Vero-E6, HEK293T, and HEK293T cells stably transfected with human Angiotensin-Converting Enzyme 2 (HEK293T-hACE2) were cultured and maintained below 14 passages as previously described [35]. The assay used an HIV-1-based SARS-CoV-2-Spike (Wuhan-Hu-1 strain) pseudotyped lentivirus, prepared as described previously [36]. Virus neutralizing activity was determined as described [35] with a few modifications for optimal resolution using HBM samples. These included dilution of defatted HBM samples in Dulbecco’s Modified Eagle Media (DMEM) containing 10% fetal bovine serum (FBS), 1% penicillin/streptomycin, and 2 mM L-glutamine (complete DMEM; cDMEM) and preparation of a 10-point 2-fold dilution series in a 96-well setup plate before incubation (at a 1:1 ratio) with SARS-CoV-2-Spike pseudovirus (1×10^5^ relative luminescence units [RLU]/mL). The remainder of the assay was as previously described [35]. The concentration of HBM needed to inhibit pseudovirus entry by 50%, termed IC_50_, was established using the neutcurve Python package (https://jbloomlab.github.io/neutcurve/).

### Statistical Analysis

Linear regressions of dose-response curves were compared through analysis of covariance and graphed through GraphPad Prism (Version 10.3.0). All other statistical analyses were performed on Rstudio (Version 4.4.1). Titers were first log-transformed and compared using Mann-Whitney test. Spearman’s correlations were measured for pair-wise comparison of log-transformed data. Correlations were first visualized with bubbleHeatmap (Version 0.1.1). Significance is defined as *P*<0.05. R code used available at: https://github.com/PMilletich/HBM_Covid19_assay.

## RESULTS

### Optimization of Multiplex Assay to Measure SARS-CoV-2 IgG and IgA in HBM

**Figure 1.**
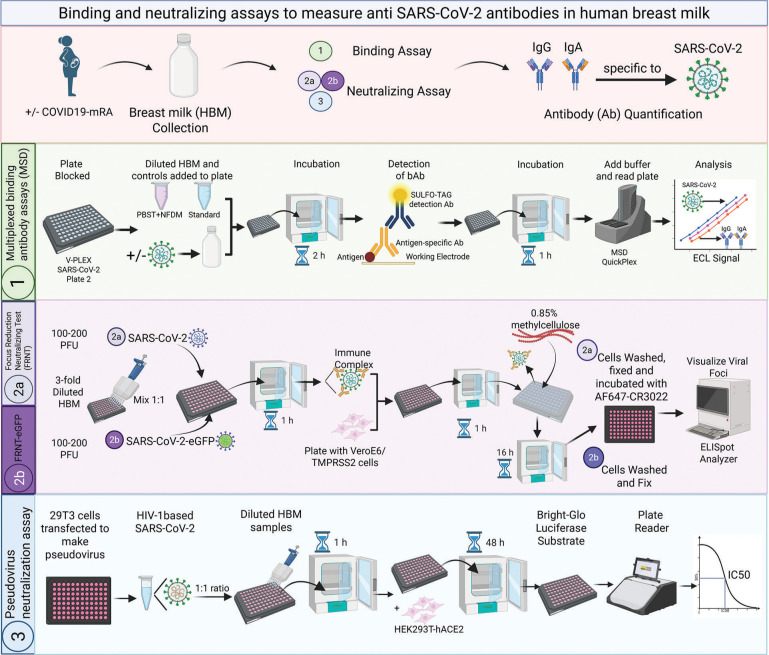
**Schematic representation of the SARS-CoV-2 multiplex binding assays and virus neutralization assays applied to human breast milk.** Created with BioRender.com PFU, plaque forming units

We developed and optimized multiplex ECLIAs for quantification of IgG and IgA specific for SARS-CoV-2 Spike, RBD, and N proteins in HBM (Figure 1). The assays were based on MSD V-PLEX SARS-CoV-2 Panel 2, which had been previously validated by the manufacturer and others for the quantification of serum antibodies [37]. Antibody titers were reported in BAU/mL referenced to the 1^st^ WHO International Standard for anti-SARS-CoV-2 Ig [23]. The optimal re-agents and assay procedures were selected as those that produced maximal signal-to-noise ratios and the widest linear analytical ranges. The established assays were then evaluated, and performance was characterized.

#### Standard curve, parallelism, dilutional linearity

**Figure 2.**
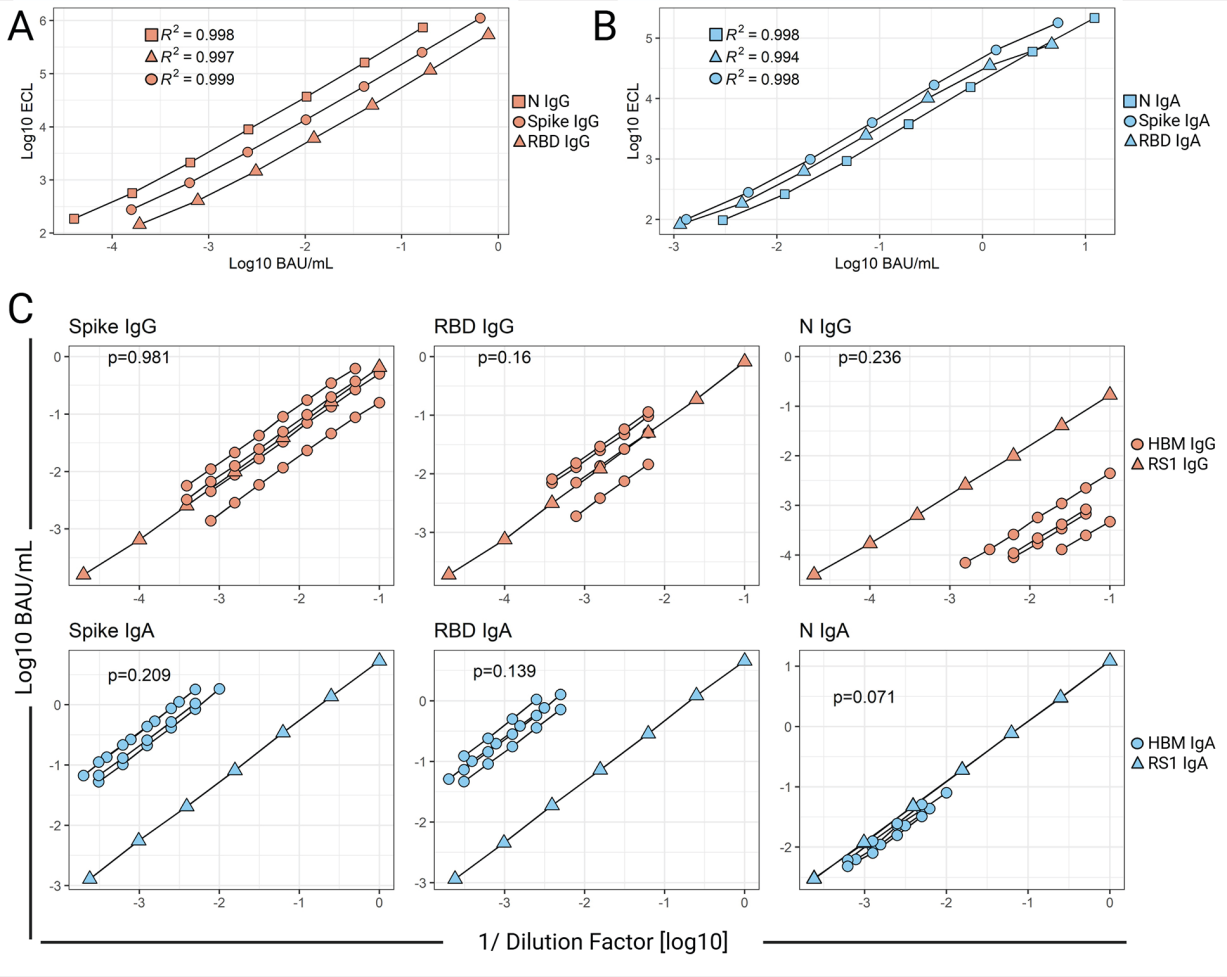
**Parallelism of SARS-CoV-2 IgG and IgA Reference Standard and human breast milk (HBM) dose-response curves.** SARS-CoV-2 Spike protein-, Spike Receptor Binding Domain-(RBD), and Nucleocapsid protein-(N) specific-IgG (A) or IgA. (B) Reference Standard 1 (RS1) curves as measured by the optimized ECLIAs. Data represent 4-parameter logistic (4PL) regression curves of electrochemiluminescent signal (ECL) vs concentration (BAU/mL) of mean values (log-transformed) from at least 7 RS1 tests in different plates. RS1 curve parameters are provided in Table 1. (C) SARS-CoV-2 antibody concentrations (BAU/mL) measured in serially diluted HBM and RS1. Data represent linear regression curves of IgG and IgA calculated concentrations vs sample dilution factor (both log-transformed). The slopes of HBM and RS1 linear regressions were compared by Analysis of Covariance; two-tailed *P*-values are shown.

Tested in serial dilutions, the assay reference standard, RS1, produced 4PL regression curves for each of the antibody specificities with acceptable R^2^ values (≥0.98), excellent linearity (recovery ~100%), and wide dynamic ranges spanning ~4 logs (Figure 2A-B, Table 1). The extended analytical range is convenient as it allows the measurement of bAb specific for multiple antigens in specimens with vastly different antibody content using a single dilution, thus minimizing repeat testing.

**Table 1.** Binding Assay Parameters for Reference Standard and Controls.

Parameter	Analyte					
	Spike IgG	RBD IgG	N IgG	Spike IgA	RBD IgA	N IgA
**Reference Standard 1**						
Concentration (BAU/mL)	6.49	7.89	1.65	5.39	4.69	12.2
Coefficient of determination (R^2^)	0.99	0.99	0.99	0.99	0.99	0.99
Linearity (average % recovery)	100.12	100.26	100.11	100.04	100.03	100.07
Minimum required dilution (MRD)	1:4	1:4	1:4	1:8	1:8	1:8
Analytical range (BAU/mL)						
Lower limit of detection (LLOD)	0.00044	0.00095	0.00011	0.0038	0.012	0.0067
Lower limit of quantification (LLOQ)	0.0018	0.0038	0.00043	0.032	0.088	0.053
Upper limit of detection (ULOD)	0.631	0.816	0.189	3.10	1.56	5.55
**Assay controls and precision**						
**Positive control 1**						
Assigned concentration (BAU/mL)	1.83	1.88	0.005	52.38	6.89	6.16
Intra-assay (%CV)	1.48	2.64	5.78	10.88	3.60	8.14
Inter-assay (%CV)	2.94	4.07	4.96	11.76	9.49	13.97
**Positive control 2**						
Assigned concentration (BAU/mL)	5.74	6.54	0.04	14.1	10.33	9.76
Intra-assay (%CV)	2.24	2.20	3.85	14.61	3.43	3.81
Inter-assay (%CV)	4.97	5.87	4.65	11.00	9.71	6.64
**Negative control**						
Average concentration (BAU/mL)	–	–	–	1.22	0.29	2.88
Intra-assay (%CV)	–	–	–	6.61	9.09	10.13
Inter-assay (%CV)	–	–	–	9.02	14.91	16.65

Reference Standard 1 (RS1) SARS-CoV-2 Ab concentrations were assigned by the manufacturer (Meso Scale Discovery, MSD). Coefficient of determination (R^2^) was averaged for seven individual RS1 curves; % recovery averaged from all points in the standard were within 80% to 120%. RBD, Spike Receptor Binding Domain; N, Nucleocapsid; BAU, binding antibody units; %CV, coefficient of variation

The similar binding profile of antibodies in the RS1 and HBM was confirmed through analysis of log-transformed bAb concentration vs sample dilution linear regression curves (Figure 2C). For each antigen and antibody isotype, no differences were observed between the slope of averaged regression curves of RS1 and those of multiple HBM samples (*P>*0.05). The demonstration of parallelism supported the use of RS1 to calculate SARS-CoV-2-specific IgG and IgA concentrations in HBM.

The multiplex ECLIAs demonstrated adequate linearity within the established detection range as evidenced by the ~45-degree slopes of the linear regressions of expected vs calculated antibody concentration (averaged slopes >0.98) and close alignment of data points, R^2^ >0.99, and Sy.x <0.02 (Supplementary Figure 1). Dilutional linearity was further confirmed through acceptable recovery of diluted samples from multiple runs, all falling within 80% to 120% (data not shown).

#### Matrix effects, minimum recommended dilutions, and limits of quantification

Breast milk is a complex tissue, and therefore, it was important to confirm the absence of matrix effects. Acceptable linearity and adequate IgG and IgA recovery was attained at 1:4 and 1:8 dilutions, respectively, and were therefore selected as the MRDs for their specific assays. The LLOQs for HBM bAb measurements were informed by the manufacturer’s LLODs and the established MRDs (Table 1).

#### Assay controls and precision

Three controls (3 positive and 1 low/negative) were identified and utilized for precision analysis. Spike-, RBD-, and N-IgG and IgA concentrations were assigned from >50 replicate tests obtained through multiple runs and operators (Table 1). The IgG reactivity in the negative control was below the LLOD, and therefore, ECL signals, instead of concentrations, were averaged, and upper ECL limits were set at the mean + 3 SD. Intra- and inter-assay precision were evaluated through the data generated to establish the bAb concentration of the controls. The intra-assay variability ranged from 1.48% to 14.61% CV, whereas the inter-assay was marginally higher, with CVs ranging from 2.94% to 16.65%. The uniformity and consistency of the HBM controls tested on multiple occasions, within and across assay runs, and involving different days and operators, confirmed the reliability of the assay results. Taken together, the results described above demonstrate the adequacy of the multiplex ECLIAs for quantification of SARS-CoV-2 IgG and IgA in diluted HBM.

#### SARS-CoV-2 Spike and Nucleocapsid IgG and IgA positivity cutoff

ROC analyses were used to determine HBM SARS-CoV-2 Spike and N antibody cutoff positivity to contextualize the data obtained (ie, Spike antibodies indicative of vaccination and/or infection, and N antibodies indicative of virus exposure/infection) (Supplementary Figure 2). Spike positivity cutoffs were established from pre-pandemic HBM (n=50) and post-pandemic HBM from mothers vaccinated with or without self-reported infection (n=236). Nucleocapsid positivity cutoffs were established from pre-pandemic HBM (n=50) and post-pandemic HBM from vaccinated mothers with self-reported COVID-19 infections prior to sample collection (n=27).

**Figure 3.**
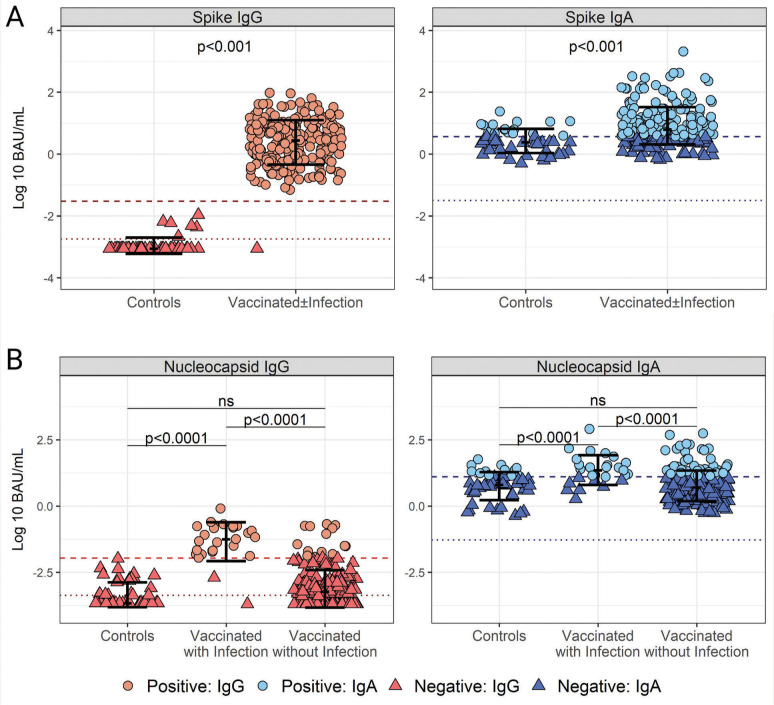
**Cutoff values for SARS-CoV-2 Spike (S)- and Nucleocapsid (N)-specific IgG and IgA positivity in human breast milk (HBM).** (A) Distribution of SARS-CoV-2 Spike IgG (left) and IgA (right) titers in pre-pandemic (n=50) and post-pandemic HBM from women vaccinated with and without self-reported infection (n=236) based on positivity cutoffs: 0.03 BAU/mL for IgG and 3.7 BAU/mL for IgA determined by Receiver Operating Characteristics (ROC) and Area under the ROC curve (AUC) (Supplemental Figure 1). (B) Distribution of SARS-CoV-2 N IgG (left) and IgA (right) titers in prepandemic (n=50) and post-pandemic HBM from women vaccinated with self-reported infections (n=27) determined by ROC and AUC analysis: 12.75 BAU/mL for IgA and 0.011 BAU/mL for IgG (Supplemental Figure 2). Data represent individual titers, mean antibody concentrations (bar), and interquartile ranges. Positivity cutoffs (dashed lines) and the lower limit of quantification (LLOQ) (dotted lines) are shown. Differences between groups determined by the Mann-Whitney test and *P*-values are indicated.

The cutoff value for Spike IgG was 0.03 BAU/mL, with 100% specificity and sensitivity and an AUC of 0.9974 (Supplementary Figure 2A). There was an obvious difference in IgG positivity between pre-pandemic and post-SARS-CoV-2 vaccination HBM samples (Mann-Whitney test, *P*<0.0001) (Figure 3A). The majority (44/50, 88%) of the pre-pandemic HBM Spike IgG titers were below the LLOQ (0.0018 BAU/mL), and the remaining six fell below the positivity cutoff of 0.03 BAU/mL. All but one (235/236, 99.6%) post-vaccination HBM sample had Spike IgG titers above the LLOQ and the positivity cutoff. The median post-vaccination IgG titer, 2.81 BAU/mL, was ~2 logs above the positivity cutoff.

The Spike IgA cutoff was determined to be 3.7 BAU/mL with 66% specificity, 71% sensitivity, and an AUC of 0.7508 (Supplementary Figure 2B). Both pre-pandemic and post-SARS-CoV-2 vacci-nation HBM exhibited IgA titers above the LLOQ; with 34% (17/50) and 71% (168/236), respectively, being above the positivity cutoff. Despite a visible overlap in individual IgA titers between the groups, the median IgA concentration in HBM post-vaccination, 6.28 BAU/mL, was significantly higher than that of the pre-pandemic controls, 2.47 BAU/mL (Figure 3A).

Nucleocapsid titers were measured to determine differences in natural infections, as this antigen was absent in the mRNA COVID-19 vaccines. The N IgG positivity cutoff, determined to be 0.011 BAU/mL, had 100% specificity, 93% sensitivity, and an AUC of 0.959 (Supplementary Figure 2C). The majority, ie, 60% (30/50) of pre-pandemic HBM samples and 45.9% (96/209) of HBM from vaccinated women without self-reported infections were below the LLOQ (0.00043 BAU/mL), while only 3.7% (1/27) of post-pandemic HBM samples with self-reported infection fell below the LLOQ. Importantly, none of the pre-pandemic HBM samples (0/50) and only 6.69% (14/209) of HBM from vaccinated mothers without self-reported infection had N IgG titers above the positivity cutoff, 0.011 BAU/mL, while 92.6% (25/27) of HBM samples from vaccinated mothers with self-reported infection were above the N IgG positivity cutoff (Figure 3B).

The N IgA positivity cutoff was 12.75 BAU/mL with 70% specificity, 76% sensitivity, and an AUC of 0.784 (Supplementary Figure 2D). All N IgA titers were above the LLOQ, 0.053 BAU/mL. Only 24% (12/50) of pre-pandemic HBM samples and 22.9% (47/209) of the HBM from vaccinated individuals without self-reported infection were above the N IgA cutoff, while in most of the post-pandemic HBM samples with self-reported infection, 70.4% (19/27) were above the N IgA positivity cutoff.

There were no differences in the distribution of N IgA and IgG when comparing pre-pandemic and post-pandemic HBM samples with no reported COVID-19 infection (Mann-Whitney, *P*>0.05), whereas titers in these 2 groups were lower than those of post-pandemic samples with self-reported COVID-19 infection (Mann-Whitney, *P*<0.0001).

#### SARS-CoV-2 binding and neutralization activity in breast milk

**Figure 4.**
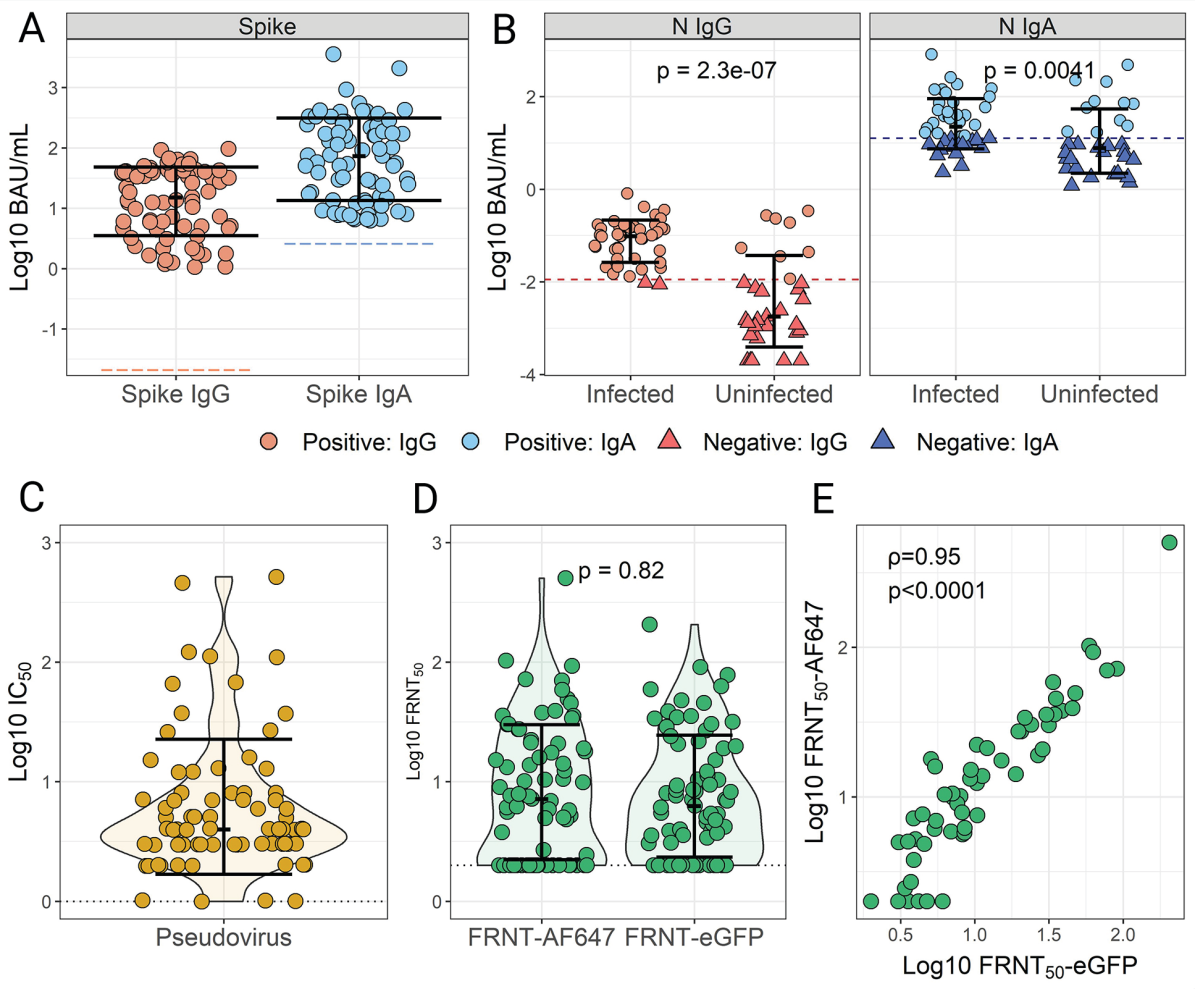
**SARS-CoV-2 IgG and IgA titers and virus neutralization activity in human breast milk (HBM) samples from vaccinated mothers**. SARS-CoV-2 Spike (A) and nucleocapsid protein (N) (B) IgG and IgA were measured in 75 HBM samples from women vaccinated with or without self-reported infection prior to sample collection. N antibody titers were grouped by self-reported SARS-COV-2 infections. Positivity was determined based on established cutoffs (Figure 4 and Supplemental Figure 2). The same HBM samples were tested for virus-neutralizing activity using a pseudovirus assay (C) and live virus focus reduction neutralization tests (FRNT) (D) using two different readouts: AF647-conjugated SARS-CoV-2 Spike antibody (FRNT_50_-AF647), and a recombinant infectious virus expressing eGFP (FRNT_50_-eGFP). Data indicate individual titers; bars show medians and interquartile ranges. (**E**) Correlation of neutralizing SARS-CoV-2 activity in HBM samples determined by FRNT methods. Spearman’s rank-order correlation (ρ) and *P*-values values are indicated.

SARS-CoV-2 bAb levels and neutralization activity were measured in a subset of 75 HBM samples from the MOMI-Vax (21-0004) study that had a broad range of anti-Spike IgG and IgA titers ≥100 AU/mL. All samples (75/75) were positive for both Spike IgG and IgA based on the established cutoffs (Figure 4A). More than half of the participants (44/75, 58.6%) self-reported COVID-19 infections before the sample collection date, of which 95.4% (42/44) were positive for N IgG, and 63.6% (28/44) for N IgA (Figure 4B). Of those with no reported COVID-19 infections, 74.2% (23/31) were negative for N IgG, and 64.5% (20/31) were negative for N IgA.

The capacity of HBM antibodies to neutralize SARS-CoV-2 in the same sample set was evaluated using live and pseudovirus neutralization assays. Pseudovirus neutralization titers ranged from 1-518 IC_50_, with a median response of 22.50±6.93 standard error of mean (SEM) (Figure 4C). In addition, 2 live virus neutralization assays based on the SARS-CoV-2 D614G variant were conducted using either the original live virus isolate with plaques detected by an Alexa Fluor-647-conjugated SARS-CoV-2 Ab or an GFP-expressing SARS-CoV-2 D614G-derived infectious clone. Similar HBM neutralization activity was observed regardless of the assay format (Mann-Whitney, *P*=0.82) (Figure 4D). The majority of HBM samples tested against the D614G isolate (68%, 51/75) or the GFP-expressing D614G (76%, 57/75) exhibited neutralizing titers above the limit of detection and comparable median values, 6.28±3.11 SEM and 7.17±6.94 SEM, respectively. There was a strong correlation between virus neutralization antibody titers (FRNT_50_) determined using the live virus isolate or the eGFP-clone (ρ=0.95, *P*<0.0001) (Figure 4E).

#### Assay concordance

**Figure 5.**
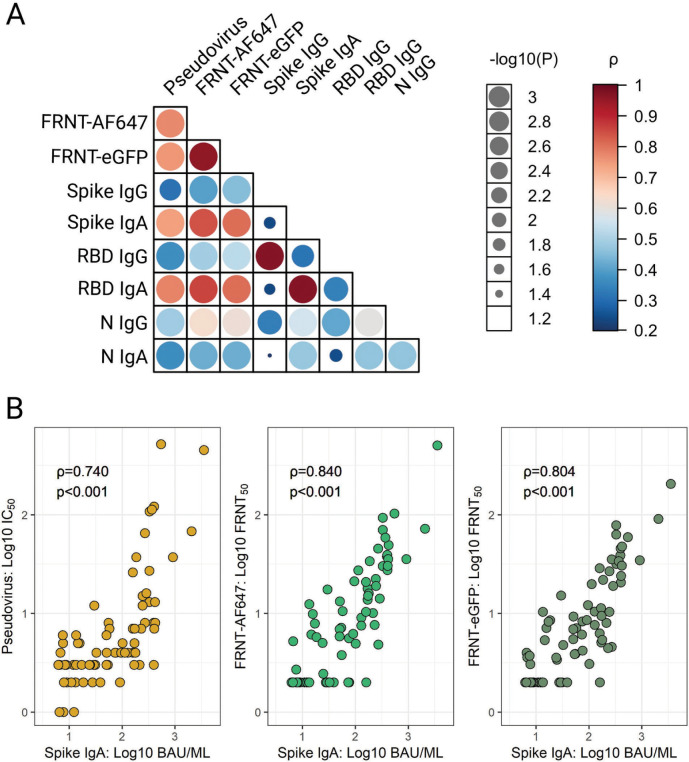
**Concordance between SARS-CoV-2 IgG and IgA titers and virus-neutralizing activity in human breast milk (HBM).** (A) Agreement between SARS-CoV-2 Spike-, Spike receptor binding domain (RBD)- and nucleocapsid (N)-specific IgG and IgA titers and virus neutralization activity measured by pseudovirus and live virus focus reduction neutralization tests (FRNT-AF647 and -eGFP) in the 75 HBM samples from vaccinated±infected women as described in Figure 4. Data were analyzed using Spearman’s rank-order on log-transformed data. (B) The most salient correlations are shown in the lower panel graphics. Data represent Spearman’s correlation coefficients (ρ) for pairwise comparisons.

Agreements between neutralization methods were examined through correlative analysis. Despite the differences in methodology and heterogeneity of HBM samples, a strong and significant association (*ρ>*0.75, *P<*0.001) was observed between both the live SARS-CoV-2 and pseudovirus neutralization titers (Figure 5A). Spike- and RBD-specific IgG titers were significantly (*P*<0.01) but modestly correlated with the virus neutralization activity, with Spearman’s ρ ranging from 0.31-0.53 (Figure 5A). Alternatively, HBM Spike- and RBD-specific IgA titers showed the highest correlations with neutralization titers with Spearman’s ρ values ranging from 0.74-0.86 (*P*<0.001). The strong associations between Spike-specific IgA and virus-neutralizing activity is further illustrated in Figure 5B. Significant but weaker correlations were found between virus neutralization and N IgA (*P*<0.001, ρ min-max: 0.37-0.43) and IgG (*P*<0.001, ρ min-max: 0.49-0.63) (Figure 5A).

## DISCUSSION

This manuscript reports the development and optimization of high-throughput, multiplex ECLIAs for quantitative measurement of IgG and IgA specific to SARS-CoV-2 Spike, RBD, and N protein in human milk. The assays were based on the MSD V-PLEX SARS-CoV-2 platform, which had been used extensively in the evaluation of COVID-19 vaccines. Correlates of protection for serum antibody levels have been established based on data generated with this methodology [38]. The optimal experimental conditions were identified, and analytical ranges and quantification limits were established for the application of the ECLIA kits to HBM. Both the IgG and IgA assays performed adequately with regard to sensitivity, repeatability, precision, linearity, parallelism, and matrix effects. The analytical characterization described herein demonstrated the adequacy of this platform for HBM bAb quantification and the reliability of the results obtained.

A variety of assays have been utilized to interrogate HBM antibodies against SARS-CoV-2 [5]. However, procedural details are often lacking, and there is minimal information on the analytical features of these assays. Data are typically reported in arbitrary units, which cannot be compared across laboratories and studies [39]. Our assays employ a rigorous procedure for antibody quantification, involving a detailed quality control process and the use of a calibrated standard curve to facilitate the comparison of SARS-CoV-2 immunogenicity readouts across studies [40].

Mothers who received COVID-19 vaccines in the MOMI-Vax study (for which these assays were set up) were eager to learn whether their breast milk contained antibodies that could benefit their infants. To discern vaccine-induced immunity, thresholds of SARS-CoV-2 Spike antibody positivity were determined based on quantitative measures of IgG and IgA in pre- and post-pandemic, post-vaccination HBM samples. The Spike IgG reactivity was clearly distinguished in post-pandemic samples with near perfect sensitivity and specificity. In contrast, IgA reactivity in pre- and post-pandemic HBM overlapped. The same exercise was applied to N antibodies to discern immunity derived from infection. Similarly to that observed for Spike IgG, an N-specific IgG cutoff allowed for explicit segregation of individuals with prior infection, whereas the IgA profile was less defined. The lower sensitivity and specificity of the IgA cutoff values are attributed to the polyreactive nature of mucosal IgA. Secretory IgA in HBM has been shown to cross-react with other human coronaviruses [15, 41, 42], whereas in general, higher specificity observed for SARS-CoV-2 IgG [17]. The broader reactivity of HBM IgA [17], as compared to IgG, is consistent with a wider range of regulatory and immune functions at the mucosal interface [43]. The more precise segregation of SARS-CoV-2 immunity based on Spike IgG in HBM was not surprising, considering that IgG is the main byproduct of parenteral COVID-19 vaccination. The higher definition of HBM N IgG in discriminating prior infection, as compared to IgA, was unexpected, given that exposure to SARS-CoV-2 triggers a mucosal response, assumed to evoke mainly IgA. This observation emphasizes the importance of IgG in the development and deployment of mucosal immunity [44]. Even though the lactating mothers might have experienced subclinical infections, the N positivity segregation based on our established thresholds aligned remarkably well with their self-reported prior infection.

Another goal of our study was to ascertain correlative associations between binding antibody levels and neutralizing activity of HBM against SARS-CoV-2. A strong correlation was found among neutralizing titers measured by the 2 live-virus assays and the pseudovirus assay, which upholds the virus-blocking capacity and putative protective contribution of HBM. Moreover, a strong association was observed between SARS-CoV-2 Spike and RBD IgA and live virus neutralization activity. There was a slightly weaker, yet still pronounced, correlation between SARS-CoV-2 Spike IgA and pseudovirus IC_50_ titers. The correlations between Spike IgG and neutralizing titers were moderately weak. Conversely, N-specific IgG concentrations aligned more closely with neutralization titers.

While virus neutralization could be attributed to a variety of components in breast milk, the strong association with HBM IgA levels is consistent with virus-blocking activity that is primarily antibody-mediated. Consistent with our findings, others have found sIgA titers in HBM to correlate with live virus-neutralization activity [19, 45].

Although comparatively lower, HBM IgG levels were still significantly associated with HBM neutralization activity in our study, particularly IgG specific for SARS-CoV-2 N, implying that albeit weaker, IgG may still contribute to mucosal viral neutralization [46].

Despite the complexity of the HBM matrix and differences in assay format (eg, virus and cell substrate), we found agreement in HBM virus neutralization activity determined by live virus or by pseudovirus assay. These results were consistent with data reported by Mühlemann et al. that showed no significant differences in the performance of various neutralization assays in human sera [47].

Whether antibodies passively transferred through breastfeeding confer protection *in vivo* remains to be elucidated; dissecting immune components associated with vaccine efficacy in mothers and infants is a major goal of the MOMI-Vax study. We have shown that maternal vaccination decreased the risk of symptomatic COVID-19 in infants [3] and that booster vaccination during pregnancy enhanced the placental transfer of antibodies to the infants [22]. We have also reported strong correlations between serum and HBM Spike-specific IgG 2 months post-delivery in mothers who received primary and/or booster doses during pregnancy or postpartum [48]. The data presented in this manuscript expands this knowledge by demonstrating the presence of SARS-CoV-2 IgG and IgA associated with virus neutralization capacity in maternal milk. An important caveat of our correlative observations is that they were derived from HBM samples with high antibody content.

Rigorous and reliable assays are important to accurately describe the breadth, durability, and functionality of SARS-CoV-2-specific antibodies in HBM and other mucosal secretions. The methods described herein for quantification of SARS-CoV-2 bAb and anti-viral function can be applied to other secretory tissues (nasal and oral fluid) and would be useful for understanding mucosal immunity against other pathogens, including those emergent and re-emergent. The knowledge generated can guide vaccine development and implementation.

## Data Availability

Raw data files will be shared upon request to the corresponding author. No novel materials were generated during this project.

## SUPPLEMENTARY DATA

Supplementary materials are available at the *Pathogens and Immunity* website. Supplementary data may be provided by the authors to benefit the reader. Supplementary data are not copyedited and are the sole responsibility of the authors. Questions or comments related to supplementary materials should be addressed to the corresponding author.
