# Supplementary material for: Development and Concordance of Binding and Neutralizing Assays to Determine SARS-CoV-2 Antibody Activity in Human Milk
Source: Pathog Immun. 2025 May 22;10(2):97–121. doi: 10.20411/pai.v10i2.799 (PMC12139606; doi:10.20411/pai.v10i2.799)
Supplement: Supplementary Figures [file pai-10-097-s01.pdf]

PATHOGENS AND IMMUNITY
In Association with Case Western Reserve University and The Richard J. Fasenmyer Foundation

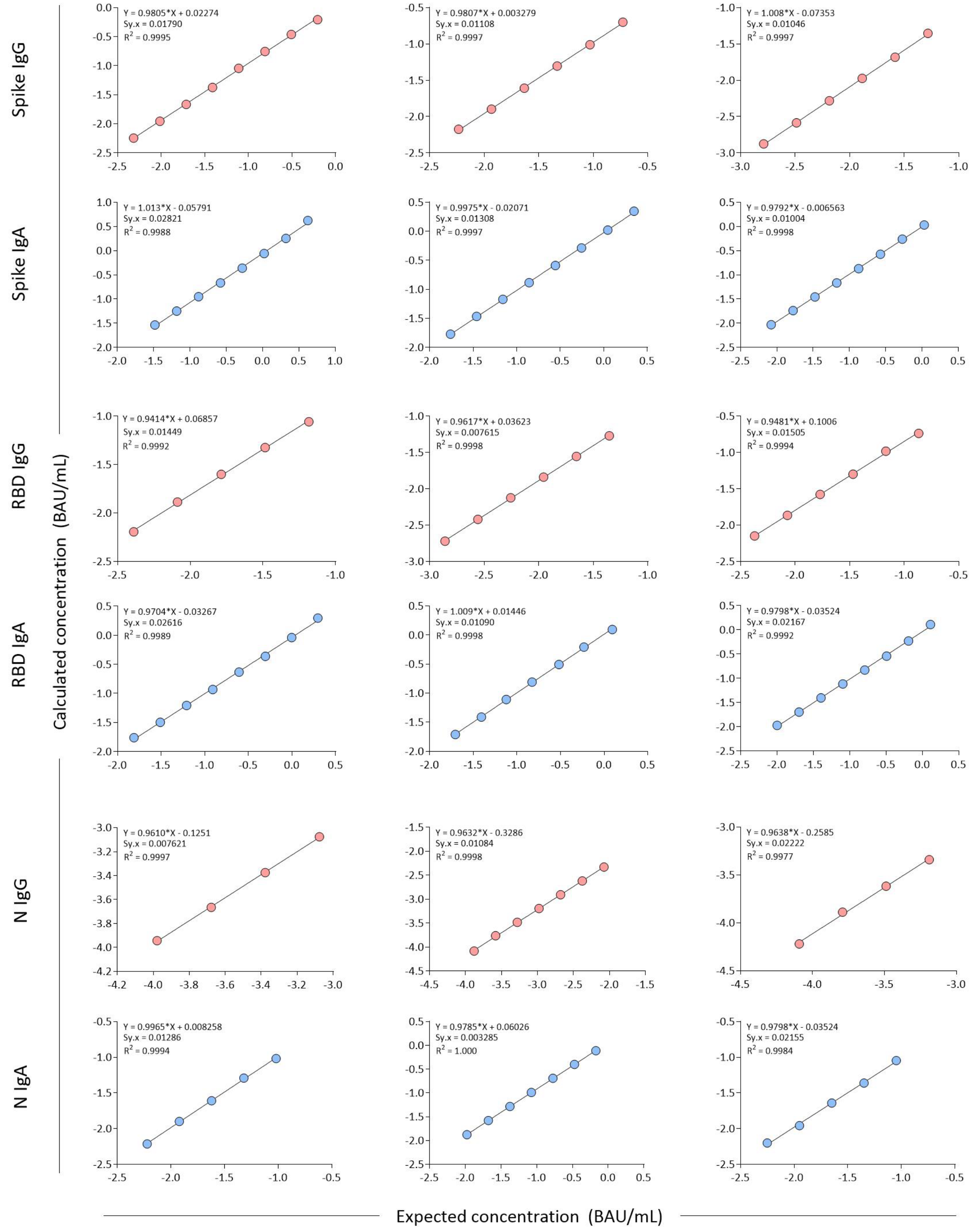

Supplemental Figure 1. Dilutional linearity of breast milk samples. SARS-CoV-2 binding antibody concentrations (BAU/mL) were measured in serially diluted human breast milk (HBM) in three samples (represented by columns). Data represent linear regression analyses of spike, spike receptor binding domain (RBD), and nucleocapsid protein (N) immunoglobulin (Ig) G and IgA calculated concentrations vs. expected concentrations (both log-transformed). The linear regression equations, Sy.x, and R^2 are indicated.

Shriver MC, Milletich PL, Moreno A, Larsen SE, Posavad CM, Berube BJ, Wali B, Ellis M, Manning K, Moore KM, Zhu Z, Grewal N, Cadena IA, Cardemil CV, Munoz FM, Neuzil KN, Coler RN, Suthar MS, Pasetti MF, and the MOMI-Vax Study Group. Development and Concordance of Binding and Neutralizing Assays to Determine SARS-CoV-2 Antibody Activity in Human Milk. *Pathogens and Immunity*. 2025;10(2):97–121. doi: 10.20411/pai.v10i2.799

PATHOGENS AND IMMUNITY
IN ASSOCIATION WITH CASE WESTERN RESERVE UNIVERSITY AND THE RICHARD J. FASENMYER FOUNDATION

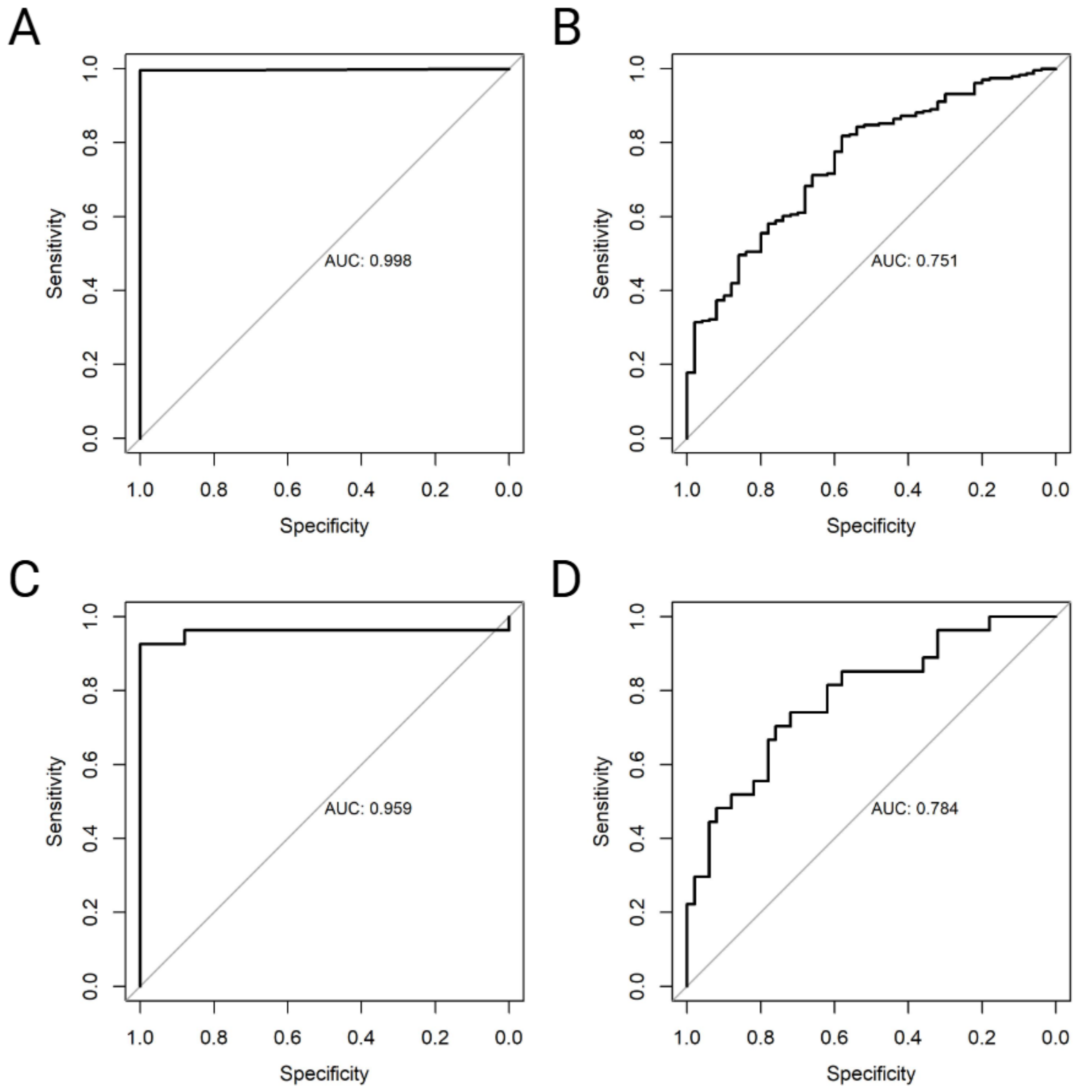

Supplemental Figure 2. Establishment of cutoff values for SARS-CoV-2 Spike- and Nucleocapsid (N)-specific IgG and IgA positivity in human breast milk (HBM). Spike and N IgG and IgA titers were determined by the optimized ECLIAs. Positivity thresholds were established by applying Receiver Operating Characteristics (ROC) and Area Under the ROC curve analysis of log-transformed binding antibodies concentrations. **(A, B)** Spike IgG and IgA thresholds were identified by comparing pre-pandemic HBM (n=50) and post-pandemic HBM from women who were vaccinated with or without self-reported infection (n=236). **(C, D)** Nucleocapsid IgA and IgG thresholds were established by comparing pre-pandemic HBM (n=50) against post-pandemic samples from vaccinated women with self-reported COVID-19 infections prior to breast milk collection (n=54).

Shriver MC, Milletich PL, Moreno A, Larsen SE, Posavad CM, Berube BJ, Wali B, Ellis M, Manning K, Moore KM, Zhu Z, Grewal N, Cadena IA, Cardemil CV, Munoz FM, Neuzil KN, Coler RN, Suthar MS, Pasetti MF, and the MOMI-Vax Study Group. Development and Concordance of Binding and Neutralizing Assays to Determine SARS-CoV-2 Antibody Activity in Human Milk. *Pathogens and Immunity*. 2025;10(2):97–121. doi: 10.20411/pai.v10i2.799
